# Changes in DMI, SDHI, and QoI Fungicide Sensitivity in the Estonian *Zymoseptoria tritici* Population between 2019 and 2020

**DOI:** 10.3390/microorganisms9040814

**Published:** 2021-04-12

**Authors:** Riinu Kiiker, Marite Juurik, Thies Marten Heick, Andres Mäe

**Affiliations:** 1 Department of Plant Protection, Estonian Crop Research Institute, 48309 Jõgeva, Estonia; riinu.kiiker@etki.ee (R.K.); marite.juurik@etki.ee (M.J.); 2 Department of Agroecology, Aarhus University, 4200 Slagelse, Denmark; thiesm.heick@agro.au.dk

**Keywords:** septoria tritici blotch, fungicide target proteins, CYP51 gene, azoles

## Abstract

*Zymoseptoria tritici* (*Zt*) populations adapt under the selection pressure of fungicides applied for disease control. The primary objective of this study was to assess fungicide sensitivity in the Estonian *Zt* population. A total of 282 *Zt* isolates from 2019 and 2020 were tested for sensitivity to azoles (DMIs; prothioconazole-desthio, epoxiconazole, mefentrifluconazole) and succinate dehydrogenase inhibitors (SDHIs; boscalid, fluxapyroxad). The efficacy of the tested fungicides varied considerably between the Estonian counties, but the *Zt* population is mainly sensitive to DMIs. Additionally, the frequencies of *CYP51* gene alterations varied; D134G, V136C, A379G, and S524T had increased, but V136A and I381V showed a moderate decrease in 2020 in comparison to 2019. Sensitivity to SDHIs was stable, but boscalid was less effective than fluxapyroxad. SdhC gene mutations C-T33N, C-T34N, and C-N86S were common, but not linked with SDHI fungicide sensitivity assay results. Otherwise, mutation B-N225I in the SdhB subunit occurred in isolates with reduced sensitivity to SDHIs. Sensitivity to strobilurins was evaluated by the mutation G143A in the *CytB* gene, which was present in nearly half of the population. The data presented confirm the ongoing evolution of fungicide sensitivity in the *Zt* population in Estonia and highlight the importance of knowledge-based decisions for optimizing anti-resistance strategies in the field.

## 1. Introduction

In the maritime zone of Europe, the primary wheat disease is Septoria tritici blotch (STB) caused by *Zymoseptoria tritici* (*Zt*). The control of STB relies on chemical fungicide applications containing active ingredients with different modes of action (MoA) since only partly resistant cultivars to this pathogen are available on the market [1,2]. The most commonly used fungicides to control STB are C-14 de-methylation inhibitors (azoles, DMIs), succinate dehydrogenase inhibitors (carboxamide, SDHIs), and quinone outside inhibitors (strobilurins, QoIs). Compounds of those three groups have been used for many years now and have efficiently mitigated the impact of STB. Azoles are the main fungicides used in STB control in Europe, which are applied for 1–4 sprays per season [3]. In Estonia, up to two applications of different MoA fungicides are used per season. There is a major variation in the field performance of fungicides across Europe, but the field efficacy of several azole fungicides (e.g., tebuconazole, metconazole) has recently declined in Europe [3,4].

The high reliance on fungicides is problematic because the consequently evolved resistance to fungicides limits our ability to control this agricultural pathogen in the field. The current Fungicide Resistance Action Committee (FRAC) recommendations for fungicide resistance management in cereals are based on limiting the amount of applications, and also the alternation and combination of fungicides using other modes of action [5]. *Zt* shows several biological traits that facilitate its adaptation under selective pressures, particularly those exerted by fungicides. Among those are a large population size, considerable genetic diversity, two modes of reproduction, and the ability to disperse over long distances via wind-borne sporangia and seed transport [6]. Many resistance cases have been described as the overexpression of target genes or efflux pumps, but the highest impact on reduced efficacy is due to substitutions in the amino acid sequence of the target protein [7]. The amino acid alterations caused by different single nucleotide polymorphisms (SNPs) change the target proteins differently, affecting the binding capacity of the fungicide to varying degrees. Several target mutations have already been described both in the lab experiments and in the field, which can lead to azole, strobilurin, and carboxamide resistance [7,8,9,10,11]. The mutation frequency and the order in which mutations accumulate can drive the evolutionary adaptation of pathogens to toxicants [12]. Resistance dynamics in pathogen populations is influenced by a combination of agronomic, biological, and climatic factors, which select pathogens for the best fit in the environment [13].

The DMI fungicides have been on the market since the late 1970s [14]. Reduced azole sensitivity of *Zt* may be conferred by different mechanisms, for instance, a combination of mutations in the *CYP51* gene [15], the overexpression of *CYP51* [16], and enhanced efflux activity due to the upregulation of ABC or MFS transporters in the membrane [17]. The most common mechanism is the accumulation of mutations in the *CYP51* gene leading to amino acid alterations in the CYP51 coded enzyme. Mutations leading to alterations D134G, V136A/C, A379G, I381V, and S524T and deletions at amino acid positions 459–460 are claimed to have the highest effect on *Zt* sensitivity to azoles [15]. To date, more than 30 different amino acid alterations (substitutions and deletions) have been identified in the CYP51 protein of European *Zt* populations [18,19]. These mutations in different combinations can affect *Zt* isolates′ level of fungicide resistance substantially [18]. During the last few years, the frequencies of the *CYP51* mutations V136C, A379G, and I381V have been relatively stable across Europe, while a clear pattern of decreasing frequencies of D134G, V136A, and S524T from west to east has been observed [4]. Due to the continuous accumulation of mutations, a reduction in the field efficacy of tebuconazole, epoxiconazole, and also prothioconazole has been observed in many European countries during the last few years [4,20,21,22,23]. The decline in the efficacy of azoles has started to accelerate, probably due to the appearance of mutation S524T in *Zt’s* field populations [4,15].

Resistance to SDHI fungicides has been reported for many plant pathogenic fungi. SDHIs inhibit fungal respiration by blocking the ubiquinone binding site, which is formed by residues of subunits B (SdhB), C (SdhC), and D (SdhD). Single amino acid substitutions in SdhB, SdhC, and SdhD have been shown to confer resistance to SDHI fungicides [24,25]. Field monitoring by FRAC members from European countries has revealed that C-T79N and C-N86S were the most frequent mutations in the last few years [26]. *Zt* isolates with reduced sensitivity were detected from Western Europe, having mutations C-T79N, C-W80S, C-N86S, B-N225T, and B-T268I each year [26]. Moreover, in 2015 the first *Zt* isolates showing a strong decrease in sensitivity to SDHIs with mutations C-H152R and D-R47W were isolated from Ireland [8]. Since then, each year isolates with moderate resistance factors and bearing the mutation C-H152R have been detected from Germany, Belgium, Ireland, the Netherlands, France, and the United Kingdom [26,27]. Amino acid exchange C-H152R is still rarely detected in the field, although, of all field mutants, it confers the highest level of resistance to all SDHIs [8,24]. Laboratory studies have demonstrated that this amino acid exchange has a substantial impact on SDHI sensitivity [28,29]. Unlike the *CYP51* mutations, haplotypes with more than one *Sdh* mutation have rarely occurred in nature yet.

In contrast to multiple DMI and SDHI resistance mechanisms, reduced sensitivity to the QoIs in most cases is conferred by the substitution of glycine by alanine at position 143 in the cytochrome b (*CytB*) gene of the pathogens’ mitochondria [30]. An additional mutation, F129L, conferring a low resistance factor, occurs very rarely in *Zt* isolates. For example, fungicides based on strobilurin (e.g., azoxystrobin) initially provided one of the best chemical solutions to manage STB, but resistance to strobilurin developed quickly. The great majority of the European *Zt* population carries the G143A mutation [23,31,32,33], making its control highly reliant on DMI fungicides.

In recent studies, we have shown that the resistance to DMI, SDHI, and QoI fungicides continues to emerge and spread in the Estonian *Zt* population. Over the period from 2014 to 2018, an increase in fungicide-resistant mutants and a shift to more complex CYP51 haplotypes were observed [23,33]. The objective of this study was to update the distribution of sensitivity to different DMI (epoxiconazole, prothioconazole-desthio, and mefentrifluconazole) and SDHI (fluxapyroxad and boscalid) fungicides commonly used in *Zt* control and to investigate the molecular resistance mechanism in *Zt* isolates collected from wheat fields in the growing seasons of 2019 and 2020. The presented data also contain a cross-resistance study comparing a *Zt* isolate’s reaction to different fungicides, including a new fungicide active ingredient mefentrifluconazole.

## 2. Materials and Methods

### 2.1. Isolate Collection

**Figure 1:**
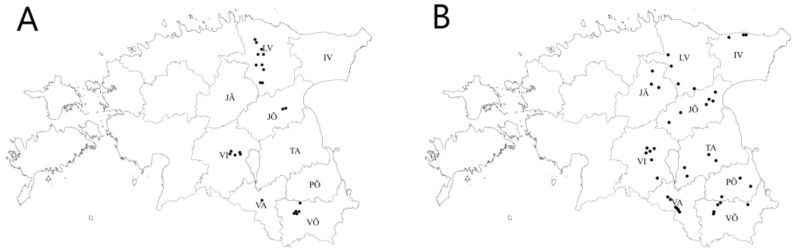
Map displaying sampling locations of the *Zymoseptoria tritici* population in Estonia in 2019 (**A**) and 2020 (**B**). Estonian counties are: IV—Ida-Viru; JÕ—Jõgeva; JÄ—Järva; LV—Lääne-Viru; PÕ—Põlva; TA—Tartu; VA—Valga; VI—Viljandi; VÕ—Võru.

In the growing seasons of 2019 and 2020, leaf samples with naturally occurring *Zt* infection were collected from the upper two leaf layers from commercial fields of winter wheat in Estonia. Samples originated from five (Jõgeva, Lääne-Viru, Viljandi, Võru, Valga) and nine (Jõgeva, Lääne-Viru, Viljandi, Võru, Valga, Ida-Viru, Põlva, Tartu, Järva) counties in 2019 and 2020, respectively (Figure 1; Table 1). STB chemical control varied in these fields from one to two fungicide applications in these fields with fungicide products.

Single pycnidium isolates of *Zt* were produced under laboratory conditions. The leaves were placed in a Petri dish on moist filter paper without prior surface sterilization. After 24 h incubation at room temperature, cirrhi from single pycnidia were transferred onto Potato Dextrose Agar and amended with 0.01% streptomycin using a sterile needle. Single spore colonies appeared after six days of incubation at 20 °C and 12 h white light/12 h darkness. Spores were conserved in 20% glycerol at −80 °C.

### 2.2. Determination of Fungicide Sensitivity

Spore suspensions were produced by scraping off 6-day-old *Zt* spores and transferring them into sterile, demineralized water. The suspensions were vortex-mixed in 10 mL Falcon tubes for 10 min for homogenization. Spore concentrations were adjusted to 2.5 × 10^4^ spores mL^−1^.

All the *Zt* isolates were tested for fungicide sensitivity for selected DMI and SDHI fungicide active ingredients except for tebuconazole, which was tested only for a subset of 32 isolates randomly selected in 2019. Epoxiconazole, tebuconazole, prothioconazole-desthio, boscalid, fluxapyroxad (all Sigma-Aldrich, St. Louis, MO, United States), and mefentrifluconazole (LGC Dr. Ehrenstorfer, Augsburg, Germany) were mixed separately with 2x Potato Dextrose Broth to obtain the following final microtiter plate fungicide concentrations (ppm): 30, 6, 1.2, 0.240, 0.048, 0.010, 0.002, and 0 for epoxiconazole; 90, 30, 10, 3.3, 1.1, 0.37, 0.12, and 0 for tebuconazole; 6, 2, 0.670, 0.220, 0.074, 0.025, 0.008, and 0 for prothioconazole-desthio; 3, 1, 0.330, 0.110, 0.037, 0.012, 0.004, and 0 for mefentrifluconazole and fluxapyroxad; 10, 3.3, 1.1, 0.370, 0.120, 0.041, 0.014 and 0 for boscalid. The same amount (100 μL) of spore suspension and fungicide solution was added to a nuncTM 96-deep well microtiter plate (Thermo Fisher Scientific, Roskilde, Denmark). Technical duplicates of each isolate were performed on the same plate, and Dutch isolate IPO323 (azole-sensitive) was included as a reference. Microtiter plates were wrapped in aluminum foil and incubated in the dark at 20 °C for six days. The plates were visually checked for bacterial and fungal contamination before the analysis in a Tecan Sunrise Microplate Absorbance Reader (Tecan, Männedorf, Switzerland) at wavelength 620 nm. The half-maximal effective concentration (EC_50_) of each fungicide was determined by non-linear regression (curve-fit) using GraphPad Prism version 9.0.2 (GraphPad Software, La Jolla, CA, United States). Resistance factors (RF) were calculated by the formula: RF = (mean EC_50_ of *Zt* population)/(mean EC_50_ of reference isolate IPO323).

### 2.3. Identifying Target Site Mutations in Fungicide Target Proteins

Genomic DNA from pure culture *Zt* isolates was extracted by the thermolysis method according to Zhang et al. [34]. *Zt* isolates were tested for mutations in *CYP51*, *CytB,* and *Sdh* genes. The presence of mutations L50S, D134G, V136A/C, G379A, I381V, and S524T in *CYP51* gene and mutation G143A in *Cytb* gene were determined using Kompetitive Allele Specific PCR (KASP) (LGC Genomics, Teddington, United Kingdom) genotyping previously described by Kildea et al. [35]. All reactions were carried out in an Applied Biosystems ViiaTM 7 Real-time PCR system machine (Thermo Fisher Scientific, Massachusetts, United States) according to the manufacturer’s protocol.

Sequences of the Sdh subunits B and C were obtained according to the protocol by Fraaije et al. [28] and Rehfus et al. [36], respectively. Primers Mgsdhbf1 and Mgsdhbr1 were applied for SdhB amplification [28] and primers KES 584 and KES 550 for SdhC amplification [36]. PCR reactions were performed in a 25 μL volume containing 10.9 μL MilliQ water, 5.0 μL 5× DreamTaq PCR buffer (Thermo Fisher Scientific, Massachusetts, United States), 100 μM of each dNTP, and forward primer and reverse primer (both 10 μM), 1 unit DreamTaq DNA polymerase (Thermo Fisher Scientific, Massachusetts, United States), and 1.0 μL DNA (approximately 10 ng μL^−1^). The amplification was performed using the following conditions: 95 °C for 5 min, followed by 35 cycles of 95 °C for 60 s, 62 °C for 30 s, 72 °C for 90 s, with a final extension of 5 min at 72 °C. PCR products were sequenced using the same forward and reverse primers using an Applied Biosystems 3730 DNA Analyzer (Thermo Fisher Scientific, Massachusetts, United States) in the Estonian Biocentre in Tartu. The sequences were compared to a reference sequence of IPO323, and target-site mutations C-N33T, C-N34T, C-T79N, C-W80S, C-N86S, C-H152R, B-N225I, and B-T268I were determined.

### 2.4. Testing for Potential Overexpression of the CYP51 and MFS1 Genes

All isolates were investigated for the presence of inserts in the *CYP51* promoter region, conferring CYP51 overexpression [16]. PCR reactions were performed in a 25 μL volume containing 10.9 μL MilliQ water, 5.0 μL 5× DreamTaq PCR buffer (Thermo Fisher Scientific, Massachusetts, United States), 100 μM of each dNTP, and forward primer Mg51-proF and reverse primer Mg51-seqR (both 10 μM), 1 unit DreamTaq DNA polymerase (Thermo Fisher Scientific, Massachusetts, United States), and 1.0 μL DNA (approximately 10 ng μL^−1^). The PCR conditions were 95 °C for 5 min, followed by 35 cycles of 95 °C for 30 s, 62 °C for 30 s, 72 °C for 30 s, with a final extension of 7 min at 72 °C.

To screen for inserts in the promoter region of the *MSF1* gene, PCR reactions were performed using primers MSF_2F and MSF_4R designed by Omrane et al. [37]. PCR reactions were set up in a 25 μL volume containing 10.9 μL MilliQ water, 5.0 μL 5× DreamTaq PCR buffer (Thermo Fisher Scientific, Massachusetts, United States), 100 μM of each dNTP, and forward primer and reverse primer (both 10 μM), 1 unit DreamTaq DNA polymerase (Thermo Fisher Scientific, Massachusetts, United States), and 1.0 μL DNA (approximately 10 ng μL^−1^). The reactions were run according to the following protocol: 35 cycles of 95 °C for 30 s, 62 °C for 30 s, 72 °C for 30 s, with a final extension of 7 min at 72 °C. All PCR reactions in this study were performed in a Mastercycler Nexus PCR Cycler (Eppendorf, Hamburg, Germany). The samples were loaded on a 1.5% agarose gel containing SYBR stain (Thermo Fisher Scientific, Massachusetts, United States) and run for 45 min at 100 V.

### 2.5. Statistical Analysis

SuperPlotsOfData Shiny app was used for visualizing the EC_50_ results [38]. Statistical analyses were performed in GraphPad Prism (GraphPad Software, La Jolla, CA, United States) and StatPlus Pro 7.3.0.0 (AnalystSoft Inc., Walnut, CA, United States). Unpaired *t*-test with Welch’s correction was applied to compare the mean EC_50_ values from the *Zt* collected in 2019 and 2020 (α < 0.05). Pearson correlation analysis for log-transformed EC_50_ values for pairs of azole fungicides was performed.

## 3. Results

The analysis of fungicide sensitivity of the *Zt* Estonian population was performed in 2019 and 2020 for 103 and 179 isolates, respectively. The *Zt* population was tested for the sensitivity of different active ingredients of DMI (prothioconazole-desthio, epoxiconazole, tebuconazole, and mefentrifluconazole) and SDHI (fluxapyroxad and boscalid) class fungicides. These active ingredients were chosen for testing as these are included in the commercial fungicide products used most often by the farmers in Estonia against STB (Table 2).

### 3.1. Status of DMI Fungicide Sensitivity

**Figure 2:**
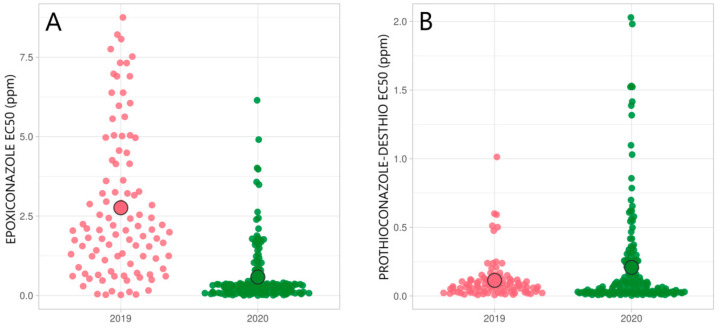

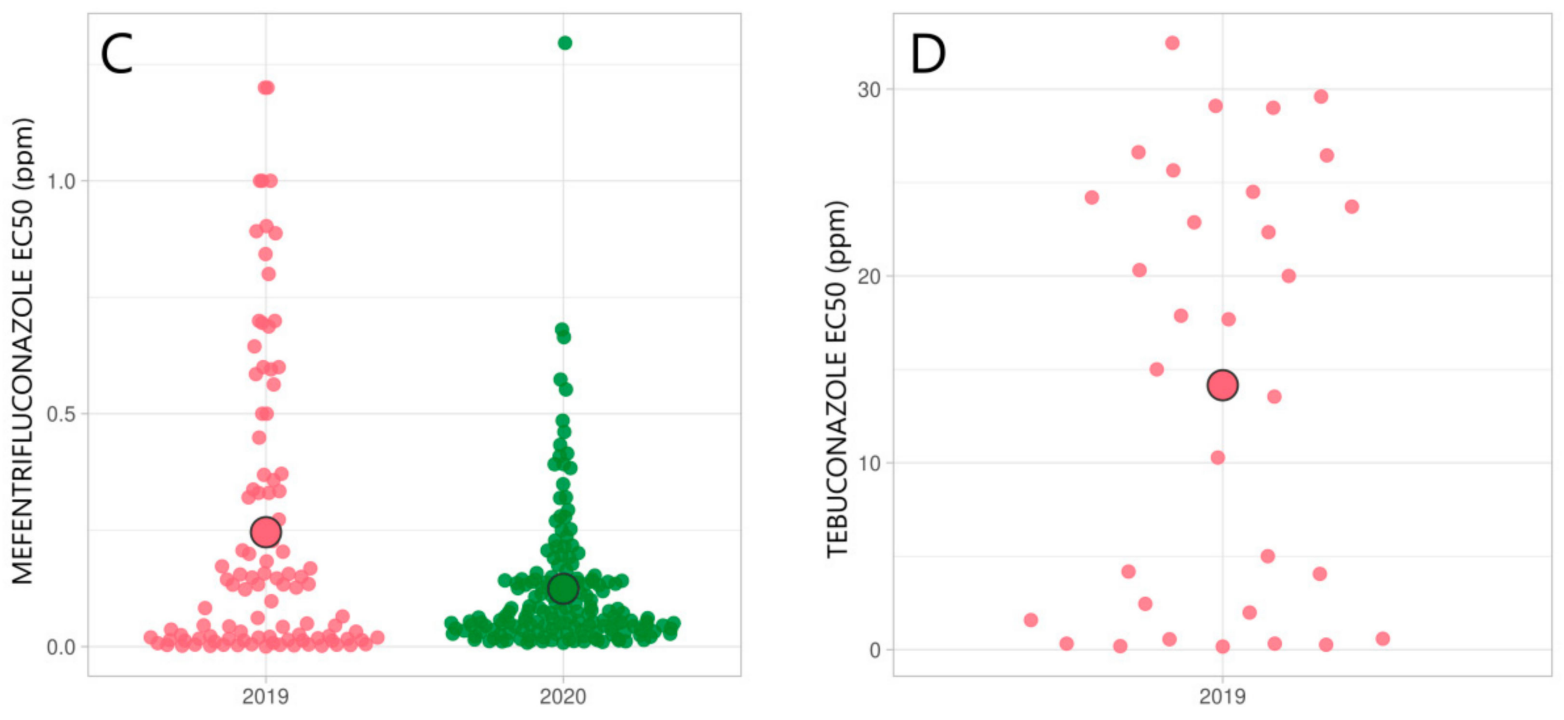
Distribution of epoxiconazole (**A**), prothioconazole-desthio (**B**), mefentrifluconazole (**C**), and tebuconazole (**D**) EC_50_ values (ppm) in Estonian *Zymoseptoria tritici* population by collection year. ○ indicates average EC_50_ value in a population.

The screening of DMI sensitivity was conducted using epoxiconazole, prothioconazole-desthio, tebuconazole, and mefentrifluconazole as representatives for DMI fungicides. From 2019 to 2020, a significant increase in sensitivity towards epoxiconazole and mefentrifluconazole was seen, with average EC_50_ values decreasing from 2.76 to 0.58 ppm (t(116) = 9.1; *p* < 0.001) and 0.25 to 0.12 ppm (t(131) = 3.63; *p* < 0.001), respectively (Figure 2). In the same period, sensitivity towards prothioconazole-desthio significantly decreased, with average EC_50_ values changing from 0.11 to 0.21 ppm (t(255) = 2.99; *p* = 0.003) (Figure 2). Tebuconazole sensitivity was tested only on a subset of *Zt* isolates from 2019; its EC_50_ value was high, 14.15 ppm on average.

Table 3 summarizes the average EC_50_ values and resistance factors (RF) for epoxiconazole, prothioconazole-desthio, and mefentrifluconazole in different counties between 2019 and 2020. From 2019 to 2020, sensitivity towards epoxiconazole increased in Jõgeva with average EC_50_ values of 2.79 ppm and 0.66 ppm, Lääne-Viru with average EC_50_ values of 2.72 and 0.72 ppm, Viljandi with average EC_50_ values of 2.98 and 0.5 ppm, and Võru with average EC_50_ values of 3.34 and 0.63 ppm, respectively. Average EC_50_ values were also low in other counties (EC_50_ values between 0.3 and 0.95 ppm), but as we did not collect *Zt* isolates from these counties in 2019, it remains unknown whether the EC_50_ values were at a low level already in 2019 or a sensitivity shift occurred in 2020.

**Figure 3:**
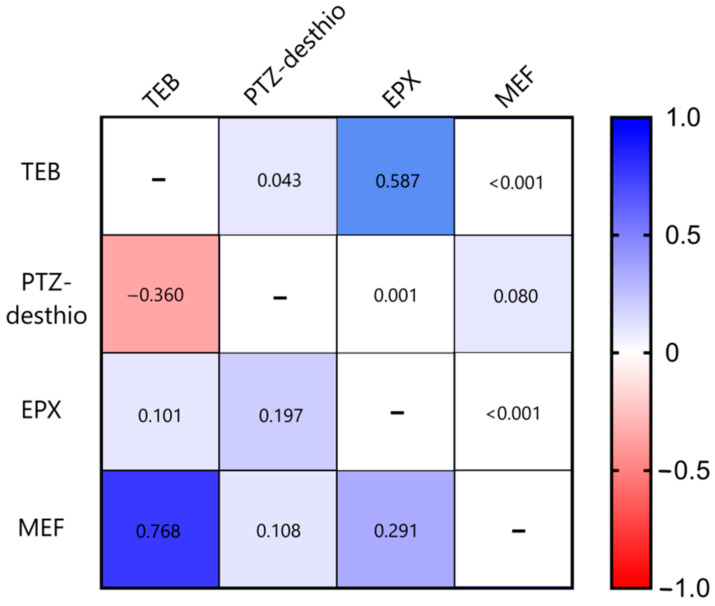
Correlation matrix between tebuconazole (TEB), prothioconazole-desthio (PTZ-desthio), epoxiconazole (EPX), and mefentrifluconacole (MEF) fungicide sensitivity in Estonian *Zt* population. Pearson correlation coefficient (*r*) values are below diagonal and *p*-values above diagonal.

Among tested isolates, twenty-two isolates from 2019 with a high EC_50_ value (>0.5) for mefentrifluconazole (Figure 2) were found from two counties, Jõgeva (avr EC_50_ = 0.41 ppm) and Lääne-Viru (avr EC_50_ = 0.3 ppm). Further analysis showed that these *Zt* isolates exhibited similar trends of reduced sensitivity to tebuconazole but not with other tested DMI fungicide active ingredients (prothioconazole-desthio and epoxiconazole). The EC_50_ values were further analyzed with Pearson correlation, which confirmed that tebuconazole and mefentrifluconazole sensitivity are highly positively correlated (r = 0.768, *p* < 0.001) (Figure 3). A significant correlation was also detected between epoxiconazole and mefentrifluconazole sensitivity (r = 0.291, *p* < 0.001), and epoxiconazole and prothioconazole-desthio (r = 0.197, *p* = 0.001). In contrast, tebuconazole and prothioconazole-desthio sensitivity were negatively correlated (r = −0.36, *p* = 0.04) in the Estonian *Zt* population.

### 3.2. Mutations in CYP51 Gene

Several point mutations in the *CYP51* gene have been associated with elevated EC_50_ values for azoles. KASP analysis of all selected isolates showed the presence of the most important mutations D134G, V136A, V136C, A379G, I381V, and S524T in the *CYP51* gene in Estonian *Zt* isolates. Mutation I381V continued to dominate throughout the region and is present in frequencies of 86–100% except for Tartu county, where this mutation was present at a low rate in 2020, 38%, respectively (Table 4). The frequencies for mutations D134G, V136A, V136C, A379G, and S524T, all of which have recently emerged in the European *Zt* population, varied considerably between counties (Table 4). On the national level, mutations D134G, V136C, A379G, and S524T had increased, but V136A and I381V showed a moderate decrease in 2020 compared to 2019 (Table 4). In both years, the most prevailing isolates (39% in 2019 and 27% in 2020) had only one mutation (I381V) in the *CYP51* gene by specific KASP analysis. The second popular mutation combination in the *CYP51* gene was A379G and I381V (14% in 2019 and 15% in 2020), followed by a combination of D134G, V136A, and I381V (15% in 2019 and 11% in 2020). Elevated EC_50_ values for epoxiconazole sensitivity (EC_50_ > 3 ppm) occurred in isolates with mutation combinations D134G, V136A, I381V, and S524T; V136A, I381V, and S524T; A379G, I381V, and S524T in the *CYP51* gene. This is the first report of significantly reduced sensitivity to mefentrifluconazole in *Zt* isolates with A379G, I381V, and S524T mutation combination in the *CYP51* gene (mefentrifluconazole EC_50_ = 0.82 ppm; tebuconazole EC_50_ = 24.75 ppm). Isolates with the combination of A379G, I381V, and S524T in the *CYP51* gene were detected only in 2019 in eight isolates from Lääne-Viru county and did not occur in the next year.

### 3.3. Analysis of CYP51 and MFS1 Promoter Region

Amplification of the *CYP51* promoter region in azole-sensitive isolate IPO323 yielded the expected wild-type 334 bp size fragment. In the Estonian *Zt* population, 14.7% (2019) and 12.5% (2020) had the wild-type promoter without inserts. However, these isolates were not necessarily the most sensitive to azole fungicides. The distribution of isolates with the 120 bp insert in the *CYP51* promoter was amplified from 15.7% (2019) and 12.5% (2020) of the *Zt* isolates. Sensitivity to tebuconazole, epoxiconazole, prothioconazole-desthio, and mefentrifluconazole had decreased for this group of isolates, with EC_50_ values being 24.75 ppm, 2.53 ppm, 0.13 ppm, and 0.34 ppm on average, respectively. Additionally, *Zt* isolates with A379G, I381V, and S524T mutation combination in the *CYP51* gene had the 120 bp insert in the *CYP51* promoter and were least sensitive to mefentrifluconazole (avr EC_50_ = 0.82 ppm) and tebuconazole (avr EC_50_ =24.75 ppm), whereas the most commonly detected insert in the *CYP51* promoter region was 866 bp long, which concurred with higher sensitivity to all tested azole fungicides, tebuconazole (avr. EC_50_ = 8.75 ppm), epoxiconazole (avr. EC_50_ = 1.21 ppm), prothioconazole-desthio (avr. EC_50_ = 0.18 ppm), and mefentrifluconazole (avr. EC_50_ = 0.13 ppm). The 866 bp insert in the *CYP51* promoter occurred in 69.6% and 75% of the 2019 and 2020 *Zt* collection, respectively.

The majority of the *Zt* isolates in 2019 (100%) and 2020 (98%) had the wild-type 486 bp long *MFS1* promoter without an insert. Only three isolates from 2020 had multi-drug resistance (MDR) type IIa 338 bp insert in the promoter region, according to Omrane et al. [39].

### 3.4. Status of SDHI Resistance and Mutations in Sdh Protein Subunits

**Figure 4:**
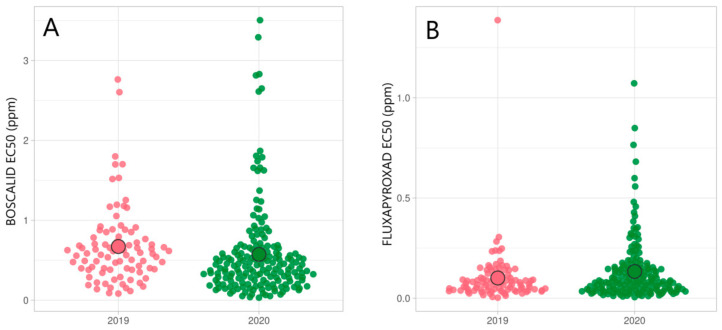
Distribution of boscalid (**A**) and fluxapyroxad (**B**) EC_50_ values (ppm) in Estonian *Zymoseptoria tritici* population by collection year. ○ indicates average EC_50_ value in a population.

The screening of SDHI sensitivity was conducted using fluxapyroxad and boscalid as representatives for SDHI fungicides. Sensitivity to both fungicides was stable in the *Zt* population in 2019 and 2020, although boscalid was less effective than fluxapyroxad (Figure 4). Boscalid sensitivity was slightly lower in 2019 (EC_50_ = 0.67 ppm) than in 2020 (EC_50_ = 0.57 ppm) (Figure 4). Average EC_50_ values for fluxapyroxad were relatively lower and stable, being 0.1 ppm in 2019 and 0.13 ppm in 2020 (Figure 4).

Table 5 summarizes the changes in EC_50_ values (ppm) and RF for boscalid and fluxapyroxad in different counties during the period 2019–2020. Sensitivity towards boscalid varied between counties, with average EC_50_ values ranging from 0.2 to 1.3 ppm in 2019 and from 0.34 to 0.84 ppm in 2020. At the same time, fluxapyroxad sensitivity was less variable between the counties than boscalid sensitivity. Average fluxapyroxad EC_50_ values ranged from 0.08 to 0.25 ppm in 2019 and from 0.08 to 0.23 ppm in 2020 in different counties.

Several point mutations in the Sdh subunits B and C have been associated with elevated EC_50_ values. In SdhC subunit mutations, C-N33T and C-N34T were identified in 65% of the isolates in 2019 and 55% of the isolates from 2020. These two mutations were found in isolates with diverse SDHI sensitivity; EC_50_ values varied between 0.03–3.51 ppm and 0.01–1.07 ppm for boscalid and fluxapyroxad, respectively. In 2019, only two isolates had serine residue (instead of asparagine) in position 86 in the SdhC subunit. In 2020, mutation C-N86S in the SdhC subunit was found more often in 9% of the *Zt* population. Although boscalid and fluxapyroxad sensitivity was high for these isolates, EC_50_ values were between 0.11–0.6 ppm and 0.04–0.1 ppm, respectively. Mutation B-N225I in the SdhB subunit was rare and detected only in 2020 in 4% of the isolates. These isolates had elevated EC_50_ values for boscalid (2.81–3.51 ppm) and fluxapyroxad (0.6–0.76 ppm). Other suggested mutations (C-T79N, C-W80S, C-H152R, B-T268I) did not occur in the Estonian *Zt* population.

### 3.5. Mutation G143A Prevalence in CytB Gene

**Figure 5:**
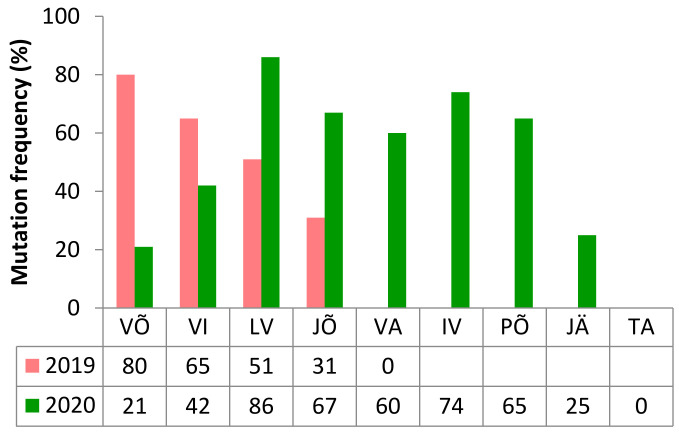
Mutation G143A frequencies in *CytB* gene by Estonian counties in 2019 and 2020. Counties are Võru (VÕ), Viljandi (VI), Lääne-Viru (LV), Jõgeva (JÕ), Valga (VA), Ida-Viru (IV), Põlva (PÕ), Järva (JÄ), Tartu (TA).

The sensitivity to QoI fungicides was determined by the presence of mutation G143A in the *CytB* gene applying KASP analysis. Overall, the G143A mutation was found in between 44 and 49% of the *Zt* population in the study years. The frequencies for G143A varied greatly among counties (Figure 5). Mutation G143A was absent in Valga county in 2019 and Tartu county in 2020. In 2019, the mutation G143A prevailed in Võru, Viljandi, and Lääne-Viru counties, and in 2020 it prevailed in Lääne-Viru, Ida-Viru, Jõgeva, Põlva, and Valga counties (Figure 5).

## 4. Discussion

Fungal plant pathogens are faced with changing environments and challenges, including overcoming host resistance in cultivars or adapting to pesticides or biological control agents. A retrospective study was performed to evaluate the changes in fungicide sensitivity in *Zt* populations collected from commercial winter wheat fields treated with fungicides for STB control. This approach was adopted as a means of studying population changes subjected to fungicide selection pressure.

In this study, 282 *Zt* isolates collected from Estonia confirmed that the sensitivity to DMI, SDHI, and QoI fungicides is highly variable in Estonia. The sensitivity towards epoxiconazole has gradually decreased from 2014 to 2018, with average EC_50_ values changing from 0.07 ppm to 2.19 ppm, respectively [23,33]. The data provided in this paper showed that the sensitivity shift continued until 2019 (EC_50_ = 2.95 ppm), but in 2020 a significant increase in epoxiconazole sensitivity was noticed (EC_50_ = 0.58 ppm), and in contrast to 2019 only a few isolates had EC_50_ values higher than 2 ppm in 2020. In contrast to epoxiconazole, the sensitivity to prothioconazole-desthio decreased significantly in 2020 (Figure 2) compared to the *Zt* population in 2018 [23] and 2019. A similar trend for epoxiconazole and prothioconazole was also observed in Denmark and Sweden, where the sensitivity towards these two DMI class fungicides shifted from 2012 to 2018, but no further sensitivity shift of DMI was seen in 2019 [21]. The sensitivity of mefentrifluconazole in the *Zt* population has been monitored in Estonia since 2019. This new azole class fungicide could be recommended for use in STB control as the efficacy remained high in the *Zt* population (EC_50_ = 0.29 ppm in 2019; EC_50_ = 0.12 ppm in 2020; Figure 2). Although mefentrifluconazole is a new active ingredient, sensitivity assay showed a broad range of sensitive and also less sensitive isolates in the Estonian *Zt* population. The data provided in this paper follow the previous findings showing some unexpected sensitivity dynamics across Europe recently [3,21] and also in different regions within single countries [20,39,40].

A pathogen population with a developed resistance to one fungicide can simultaneously resist one or several other fungicides, a phenomenon known as cross-resistance. Usually, cross-resistance appears among fungicides with the same mode of action as azoles [4,21]. In this study, tebuconazole and mefentrifluconazole sensitivity showed a high correlation, which is a matter of concern in STB control. These results were also confirmed by Heick et al. [21], who showed a very strong cross-resistance between mefentrifluconazole, difenoconazole, and tebuconazole. There is a possible pre-selection of the *Zt* population already less sensitive to tebuconazole also with highly variable mefentrifluconazole sensitivity [21]. Tebuconazole has been applied for decades for disease control, and after a decline in its efficacy [3,4], an increase in sensitivity in the *Zt* population was observed in Northern Europe [21]. In the STB control in fields, mefentrifluconazole shows high efficacy and performs much better than tebuconazole due to its higher intrinsic activity [21]. Still, given a pre-selected population, anti-resistance strategies are important not to select for those resistant haplotypes.

Besides, cross-resistance may even occur between fungicides with distinct modes of action. In field isolates of *Zt*, enhanced efflux contributes to the pathogen’s cross-resistance to several fungicides with different modes of action [37]. Applying effective disease control plays an essential role in receiving higher crop yields and producing high-quality cereals; thus, only effective fungicides with different cross-resistance patterns and with various modes of action should be used in the region [3,21,41].

The reduction in sensitivity to azole class fungicides might have slowed down in the *Zt* population either because *CYP51* is not mutating any further or the least sensitive mutation combinations have low fitness in nature (depending on weather conditions, host, fungicide pressure, etc.). Several studies have previously demonstrated that SNPs in the *CYP51* gene are the primary force behind azole resistance [7,15,17]. However, as not all alterations are equally important, single frequencies of specific *CYP51* mutations give a proper indication for the selection status of a population [7]. In 2019–2020, the most important mutations D134G, V136A, V136C, A379G, I381V, and S524T in the *CYP51* gene were present in the Estonian *Zt* population. The data presented show that mutation I381V remains the most predominant, though its frequency decreased from 100% in 2019 to 88% in 2020 (Table 4). This concurs with findings from all around Europe [3,33]. Point mutation A379G, which occurs in combination with I381V, was stable at around 20% frequency. In addition to these two mutations, we also observed a decrease in the frequency of mutation V136A from 45% in 2019 to 30% in 2020. In contrast, frequencies of D134G, V136C, and S524T continue to increase in the Estonian *Zt* population compared to previous years [23]. The most frequent combination of D134G, V136A, and I381V mutations and A379G and I381V mutations in the *CYP51* gene found in the Estonian *Zt* population is in agreement with findings from several European countries where these isolates have been established [18]. The mutations in the *CYP51* gene occur in different combinations, which may affect the pathogen’s fitness or change their reaction to fungicide applications [15].

Though the data provided in this study demonstrated the increase in sensitivity towards tested azole class fungicides, there were few cases of isolates with highly reduced fungicide sensitivity in 2019 and 2020 (Figure 2). For instance, sensitivity to mefentrifluconazole was reduced in *Zt* isolates with A379G, I381V, and S524T mutation combination in the *CYP51* gene and potential overexpression of the CYP51 protein with the 120 bp insert in the promoter region. Additionally, sensitivity to tebuconazole, epoxiconazole, and mefentrifluconazole had decreased for isolates with the 120 bp insert in the CYP51 promoter, which causes the overexpression of the *CYP51* gene affecting sensitivity to azoles [16]. Though these “high-risk” isolates have been found only in some Estonian counties (Figure 2), their presence in the field should not be underestimated as extensive or long-term use of azoles might spread azole resistance in the population further. A comprehensive study by Blake et al. [39] showed a good correlation between in vitro fungicide sensitivity assays with *Zt* isolates in the laboratory conditions and the field performance of DMI and QoI fungicides, which supported the use of laboratory assays on tracing insensitive isolates as a warning of future changes in field performance.

Distinct molecular mechanisms confer resistance to SDHI class fungicides, among which the mutations leading to amino acid substitutions in the SDH protein are the most common [29]. In this study, average EC_50_ values for boscalid were 0.67 ppm and 0.57 ppm in 2019 and 2020, respectively. In the same period, average EC_50_ values for fluxapyroxad were 0.1 ppm in 2019 and 0.13 ppm in 2020. Only two isolates surpassed the EC_50_ of 1.0 ppm of fluxapyroxad. EC_50_ values for boscalid were relatively higher than those for fluxapyroxad due to the compound’s lower intrinsic activity. Between the years 2012 and 2015, five different Sdh variants, C-T79N, C-W80S, C-N86S, B-N225T, and B-T268I, which gave low resistance to SDHI group fungicides, were reported in several European countries [26]. In Ireland, the reduced field efficacy of SDHI was explicitly correlated with a high frequency of C-T79N (S. Kildea, personal communication). Additionally, field strains collected in Ireland carrying mutation C-H152R showed high resistance to SDHIs [8]. Fortunately, none of the *Zt* isolates from Estonia carry the C-T79N or C-H152R mutation in the SdhC subunit. In a previous study carried out in 2018, no mutations in the SDH gene were detected in the Estonian *Zt* population [23]. In this study, the combination of mutations C-N33T and C-N34T was the most prevalent, with frequencies of 65% in 2019 and 55% in 2020 with variable boscalid and fluxapyroxad sensitivity. These findings are similar to the results presented by Yamashita and Fraaije [42] and Dooley et al. [8]. In addition, fluxapyroxad sensitivity was correlated with boscalid, penthiopyrad, and bixafen sensitivity [42]. The gene expression study indicated that the overexpression of an ABC/MDR transporter might contribute to the phenotype [37]. Additionally, mutation C-N86S was found in 2020, but the SDHI sensitivity results were contrasting to Rehfus et al. [36]. Additionally, B-N225I mutation occurred in 4% of the Estonian *Zt* population in 2020, which resulted in reduced sensitivity to boscalid and fluxapyroxad. We cannot rule out the possibility that the plant or fungus itself can degrade SDHIs. Our findings suggest that SDHI fungicide field applications may select for insensitive Sdh variants in the *Zt* population in Estonia. These isolates need to be further evaluated to retest sensitivity and to find out if other mechanisms were contributing to increased EC_50_ values in *Zt* isolates.

The QoIs were a highly effective class of fungicides in the mid-1990s [30]. Resistance to the QoIs in *Zt* populations occurred already at the beginning of the 2000s [31]. In most cases, resistance to the QoIs is conferred by the mutation G143A in the *CytB* gene. G143A mutants are known to have a high level of cross-resistance between different strobilurins [30]. Due to the absence of any observable fitness penalties associated with resistance, the resistant allele has rapidly spread and remained at extremely high frequencies in *Zt* populations [32,33,43]. In the Estonian *Zt* population, the mutation G143A was detected for the first time only recently in 2018 [23]. Though the QoI fungicides are still used in STB control in Estonia, the average frequency of G143A has been stable during the last few years (50% in 2018, 44% in 2019, and 49% in 2020). Still, the frequency was variable between different counties and study years (Figure 5). Maintaining the use, but in a reasoned manner in combination with other fungicide classes such as DMIs, SDHIs, and multisite fungicides, can still be recommended to manage STB in Estonia.

To conclude, the Estonian *Zt* population is sensitive to tested SDHI fungicides, although with intensive farming caution should be taken because of elevated EC_50_ values in some counties (Jõgeva, Lääne-Viru, Järva). Sensitivity to DMI fungicides is variable, but the *Zt* population is mainly sensitive to prothioconazole and mefentrifluconazole, in contrast to epoxiconazole and tebuconazole. The stable frequency of G143A mutation in the *CytB* gene in around half of the *Zt* population allows the rational application of QoI fungicides in STB control. In addition to the fungicide sensitivity of the pathogen, the regional selective pressure of fungicide applications and the intensity of local STB epidemics should be considered in disease management strategies. It would help farmers adapt their spray programs while implementing sustainable local strategies before resistance reaches fixation in the population and fungicide field performance declines. Furthermore, it is essential to continue with *Zt* population monitoring, tracing insensitive isolates, and assessing fungicide sensitivity dynamics in Estonia because, in recent years, STB has become a major disease, causing substantial wheat yield loss.

## 5. Conclusions

The intensive use of azole fungicides is driving the development of azole resistance, as observed by the accumulation of *CYP51* mutations. Azole fungicides have a critical role in STB field control in Estonia, although data from this study showed an increase in epoxiconazole and mefentriflucozole sensitivity and a reduction in prothioconazole-desthio sensitivity in the *Zt* population. Among two SDHIs, fluxapyroxad showed higher efficacy than boscalid. The stable frequency of G143A mutation in the *CytB* gene in around half of the *Zt* population allows the rational application of QoI fungicides in STB control. Despite some differences in fungicide susceptibility currently present in the *Zt* population, the rotation or mixing of fungicides may be possible to control STB.

## Figures and Tables

**Table 1:** Collection of *Zymoseptoria tritici* isolates from Estonia in 2019 and 2020.

Year	County *	No of Fields	No of Isolates
2019	JÕ	3	19
	LV	8	49
	VA	1	6
	VI	5	19
	VÕ	2	11
2020	IV	4	19
	JÕ	5	24
	JÄ	4	20
	LV	4	21
	PÕ	3	17
	TA	4	21
	VA	3	15
	VI	5	21
	VÕ	4	21
Total		55	282

* IV—Ida-Viru; JÕ—Jõgeva; JÄ—Järva; LV—Lääne-Viru; PÕ—Põlva; TA—Tartu; VA—Valga; VI—Viljandi; VÕ—Võru.

**Table 2:** Most common fungicides applied in the wheat fields in 2019–2020 in Estonia.

Commercial Product	Field Treatment (L ha^−1^)	Active Ingredients	Concentration (g ha^−1^)
Viverda (BASF)	0.8	Epoxiconazole *	40
		Boscalid *	112
		Pyraclostrobin	48
Input (Bayer)	0.4	Prothioconazole *	64
		Spiroxamine	120
Curbatur (Bayer)	0.4	Prothioconazole *	100
Tango Super (BASF)	0.6	Epoxiconazole *	50.4
		Fenpropimorf	150
Priaxor (BASF)	0.4	Fluxapyroxad *	30
		Pyraclostrobin	60

* Selected fungicide active ingredients or their metabolite for testing sensitivity in *Zt* population.

**Table 3:** Average EC_50_ values (ppm) and resistance factors (RF) for epoxiconazole (EPX), prothioconazole-desthio (PTZ-desthio), and mefentrifluconazole (MEF) of *Zymoseptoria tritici* isolates collected in Estonia 2019 and 2020 by counties.

County *	Year	EPX		PTZ-desthio		MEF	
		EC_50_	RF	EC_50_	RF	EC_50_	RF
IV	2019	NA **	NA	NA	NA	NA	NA
	2020	0.3	12	0.5	35	0.17	163
JÕ	2019	2.79	109	0.1	10	0.41	412
	2020	0.66	26	0.23	16	0.22	218
JÄ	2019	NA	NA	NA	NA	NA	NA
	2020	0.68	27	0.2	14	0.06	58
LV	2019	2.72	105	0.13	13	0.3	300
	2020	0.72	28	0.23	16	0.13	124
PÕ	2019	NA	NA	NA	NA	NA	NA
	2020	0.95	37	0.42	29	0.09	93
TA	2019	NA	NA	NA	NA	NA	NA
	2020	0.43	17	0.07	5	0.07	73
VA	2019	1.37	53	NA	NA	0.01	5
	2020	0.48	19	0.08	6	0.06	63
VI	2019	2.98	114	0.07	7	0.1	95
	2020	0.5	19	0.08	6	0.11	105
VÕ	2019	3.34	131	0.16	16	0.1	98
	2020	0.63	25	0.11	8	0.16	157
Ref. IPO323		0.026	NA	0.014	NA	0.001	NA

* Counties are: IV—Ida-Viru; JÕ—Jõgeva; JÄ—Järva; LV—Lääne-Viru; PÕ—Põlva; TA—Tartu; VA—Valga; VI—Viljandi; VÕ—Võru. ** NA—not available.

**Table 4:** *CYP51* mutation frequencies (%) in *Zymoseptoria tritici* population from Estonia in 2019 and 2020. Frequencies between 1 and 20% are indicated in green, 21–50% are yellow, 51–100% are red, missing mutations (0%) are blue.

County *	*CYP51* Mutation Frequency (%)											
	D134G		V136A		V136C		A379G		I381V		S524T	
	2019	2020	2019	2020	2019	2020	2019	2020	2019	2020	2019	2020
IV	NA **	5	NA	5	NA	0	NA	42	NA	100	NA	0
JÕ	0	38	0	38	0	25	31	8	100	92	0	33
JÄ	NA	60	NA	55	NA	0	NA	10	NA	90	NA	40
LV	12	33	33	24	22	19	33	33	100	95	20	24
PÕ	NA	29	NA	35	NA	0	NA	24	NA	100	NA	35
TA	NA	62	NA	19	NA	0	NA	10	NA	38	NA	14
VA	67	40	67	33	0	0	6	0	100	100	0	33
VI	46	62	46	48	12	14	23	48	100	86	12	19
VÕ	60	42	80	5	0	16	0	16	100	95	20	16
Average	37	42	45	30	7	10	19	21	100	88	10	24

* Counties are: IV—Ida-Viru; JÕ—Jõgeva; JÄ—Järva; LV—Lääne-Viru; PÕ—Põlva; TA—Tartu; VA—Valga; VI—Viljandi; VÕ—Võru. ** NA—not available.

**Table 5:** Average EC_50_ values (ppm) and resistance factors (RF) for boscalid (BOS) and fluxapyroxad (FLX) of *Zymoseptoria tritici* isolates collected in Estonian counties in 2019 and 2020.

County *	Year	BOS		FLX	
		EC_50_	RF	EC_50_	RF
IV	2019	NA **	NA	NA	NA
	2020	0.34	2	0.1	1
JÕ	2019	0.52	4	0.09	1
	2020	0.48	3	0.16	2
JÄ	2019	NA	NA	NA	NA
	2020	0.84	6	0.23	3
LV	2019	0.74	5	0.08	1
	2020	0.47	3	0.09	1
PÕ	2019	NA	NA	NA	NA
	2020	0.35	2	0.08	1
TA	2019	NA	NA	NA	NA
	2020	0.7	5	0.14	2
VA	2019	0.2	1	NA	NA
	2020	0.78	5	0.15	2
VI	2019	0.58	4	0.08	1
	2020	0.46	3	0.1	1
VÕ	2019	1.3	9	0.25	3
	2020	0.75	5	0.14	2
Ref. IPO323		0.144	NA	0.083	NA

* Counties are: IV—Ida-Viru; JÕ—Jõgeva; JÄ—Järva; LV—Lääne-Viru; PÕ—Põlva; TA—Tartu; VA—Valga; VI—Viljandi; VÕ—Võru. ** NA—not available.

## Data Availability

Data are available upon request.
